# hTERT-immortalized mesenchymal stem cell-derived EV treatment reduces ZIKV-induced cortical neuronal death, infection, and exosome-mediated transmission

**DOI:** 10.1128/spectrum.02524-25

**Published:** 2026-04-13

**Authors:** Kehinde Damilare Fasae, Ana Melentijevic Eckert, Girish Neelakanta, Hameeda Sultana

**Affiliations:** 1 Department of Biomedical and Diagnostic Sciences, College of Veterinary Medicine, University of Tennessee 70737, Knoxville, Tennessee, USA; 2 American Type Culture Collection (ATCC) 36576, Gaithersburg, Maryland, USA; Karolinska Institutet, Stockholm, Sweden

**Keywords:** hTERT MSC, extracellular vesicles, cortical neurons, mice, Zika virus, neuronal viability, apoptosis, GW4869, CD9

## Abstract

The application of stem cells, mesenchymal stem cells (MSCs), and exosomes/extracellular vesicles (EVs) derived from these stem cells has shown potential advancement in solving several major medical complications. Recent studies on MSC-EVs have gained focus on tissue repair, inflammation, apoptosis, fibrosis, and regenerative medicine. MSC-EVs modulate and play potential therapeutic roles in the treatment of various diseases such as cancer, neurological disorders, and viral infections. In the current study, we provide evidence that human telomerase reverse transcriptase (hTERT)-immortalized MSC-EVs possess a neuroprotective role in Zika virus (ZIKV)-infected murine cortical neurons. The American Type Culture Collection (ATCC) 2022 challenge award (call for proposals to use the product “ATCC SCRC-4000-EXM,” hTERT-immortalized MSC-EVs in new, interesting, or daring applications) afforded us the opportunity to test these hTERT-MSC-EV in ZIKV-infected neurons. We found that hTERT-MSC-EV treatment increased the cell viability of ZIKV-infected neurons and decreased the expression of apoptotic markers (Bax, caspases 3/9, and Bcl-2). In addition, hTERT-MSC-EV treatment reduced ZIKV loads in cortical neurons that correlated with increased interferon-beta and decreased TNF-alpha transcripts. Poly I:C (polyinosinic:polycytidylic acid) stimulation and treatment of uninfected cortical neurons with hTERT-MSC-EV showed an increased trend in IFN-alpha levels, but IFN-beta transcript levels were reduced. Furthermore, hTERT-MSC-EV treatment continued to reduce viral loads, even upon Poly I:C stimulation and ZIKV infection. GW4869 inhibition followed by hTERT-MSC-EV treatment revealed that exosome-mediated viral transmission is also affected in ZIKV-infected cortical neurons. Overall, this study suggests a potential therapeutic effect of hTERT-MSC-EV in ZIKV-induced neuronal death and perhaps in controlling exosome biogenesis during viral infections. This study opens new thoughts on how hTERT-MSC-EV could be proposed as a therapeutic potential in ZIKV-mediated microcephaly by enhancing cell viability of ZIKV-infected cortical neurons, inhibiting apoptosis, and reducing viral infection and exosome-mediated transmission in fetal and newborn brains.

**IMPORTANCE:**

Zika virus (ZIKV), a mosquito-borne flavivirus that vertically transmits from pregnant women to her fetus, causes microcephaly, a birth defect where newborns show smaller head circumference and brain size compared to normal healthy babies. Microcephalic newborns exhibit several developmental delays and neurological complications. There is no cure or potential therapeutics available to treat ZIKV-caused microcephaly. Human telomerase reverse transcriptase (hTERT)-immortalized mesenchymal stem cell (MSC)-derived extracellular vesicles (MSC-EVs) have been recently proposed as potential therapeutics in the treatment of various diseases, including cancer, neurological disorders, and viral infections. We hypothesized an important role of hTERT MSC-EVs in providing neuroprotective effects upon ZIKV infection in murine cortical neurons. Our study opens new thoughts on how hTERT-MSC-EV could be proposed as potential therapeutics in ZIKV-caused microcephaly, where they enhance cell viability, inhibit apoptosis, and reduce viral infection and exosome-mediated transmission/dissemination of ZIKV-infected murine cortical neurons in embryonic brains.

## INTRODUCTION

Extracellular vesicles (EVs) or exosomes are membrane-bound vesicles that mediate intercellular and intracellular communications between cells and within the tissues (1, 2). EVs can be identified through enriched markers that include CD9, CD63, CD81, TSG101, Alix, and others (1, 2). The distribution of these markers depends on the type and function of a cell. EVs in the range of 30–150 nm have been described as exosomes, which carry cellular contents such as nucleic acids (mRNA, miRNA, and DNA), proteins, and lipids across the biological membranes (1–3). Vesicular interactions play a major role in communication between neighboring cells, immune cells, or cancerous cells, thus allowing for targeted exchange of nucleic acids and proteins (4–7). Several studies have demonstrated the involvement of EVs in mediating viral infections (7–16). The composition of EVs changes during viral infections, therefore, leading to either restriction or induction of viral propagation in recipient cells (3, 7–20). Upon membrane binding and fusion with the recipient cells, EVs release functional molecules such as RNA into these cells, thereby facilitating virus transmission and spread within and between the cells (3, 7–20). Our previous studies with arthropod or neuronal EVs have shown that infectious Zika virus (ZIKV)/West Nile virus and dengue virus or Langat viral mRNA fragments and/or full-length RNA genomes were contained inside the EV lumen (7, 9, 14, 16). These EVs could transmit the viral RNAs into naive recipient cells when under certain stressful conditions, such as viral infections, thus suggesting an important role for EVs (7, 9, 14, 16).

In addition to promoting the viral spread from one cell to another, EVs could also trigger host cell antiviral response by directly modulating the immune system, blocking viral infections, and promoting antiviral activity (7, 9, 14, 16). Recently, EVs of mesenchymal stem cell (MSC-EVs) origin have gained attention due to their ability to recapitulate some regenerative effects of their mother cells (21–39). MSCs are tissue-derived multipotent non-hematopoietic adult stem cells, with low immunogenicity and regenerative capacity (21–39). During injury, repair, and disease processes, MSCs efficiently communicate with target cells by modulating their function and activity (21–40). However, ethical and safety concerns have limited the use of MSCs in clinical settings (41, 42). An increasing number of studies have reported using MSC-derived EVs, with a high success rate due to their cell-free state, targeted delivery, and high efficiency of repairability (27, 39). Like other EVs, MSC-EVs facilitate the transport of functional biomolecules such as proteins, DNA/RNA, mRNA, miRNA, and lipids between cells and have been reported to exhibit immunomodulatory and anti-inflammatory activities (1, 38, 43–45). This ability of MSC-EVs has been exploited to combat tissue injuries and chronic diseases, including tuberculosis, kidney dysfunction, cancer, neurological defects, and viral infections (21, 23, 26, 27, 29–31, 34, 36, 37, 46–49). In addition, the anti-apoptotic effects of MSC-EVs has been consistently documented (21, 29, 50, 51). Yet, the mechanisms governing MSC-EVs-mediated inhibition of apoptosis are not clearly understood. There are studies and reports demonstrating the beneficial effects of MSC-EVs against viral infections, including SARS-CoV-2, hepatitis C virus, and influenza-H5N1 viruses (33, 36, 37, 52–54). Our previous study showed that ZIKV infection kills primary cultures of murine cortical neurons on days 4 and 5 (96–120 h) post-infection (16). Few studies have evaluated the effects of neuroinvasive flaviviruses such as ZIKV on MSCs (55). However, the effects of MSC-EVs on the replication and transmission of ZIKV or neuronal cell death induced by ZIKV were not explored. We hypothesized that treatment with human telomerase reverse transcriptase (hTERT)-immortalized MSC-EVs might exert neuroprotective effects in ZIKV-infected murine cortical neurons. The American Type Culture Collection (ATCC) 2022 challenge award (call for proposals to use “ATCC SCRC-4000-EXM,” hTERT-immortalized MSC-EVs in new, interesting, or daring applications) afforded us the opportunity to test the potential therapeutic role of hTERT-MSC-EV on ZIKV-infected neurons. The hTERT technology has been well highlighted in producing the hTERT-MSC-EV that are superior in comparison to the primary MSC-EVs. A recent study reported that hTERT-immortalized MSC-EVs have potential reparative properties against damaged retinal cells similar to those observed in primary MSC-EVs (56). In the current study, we determined the effects of hTERT-MSC-EV (obtained from ATCC) on ZIKV-mediated cell death in primary cultures of cortical neurons.

## MATERIALS AND METHODS

### Isolation of murine cortical neuronal primary cultures, treatments, and infection

Primary cultures of cortical neurons were isolated from embryonic day 16 brains (female mice with E16 gestation period) and seeded at a density of 2 × 10^5^ per well of a 12-well plate (pre-coated with 5 µg/mL of poly-L-lysine). Neurons were grown in complete neurobasal media containing 10% heat-inactivated fetal bovine serum and cultured according to the methods previously described in our publications (7, 16). The American Type Culture Collection product “ATCC SCRC-4000-EXM” was received as part of the ATCC Innovation Challenge award. As expected, hTERT-MSC-EV met well-established quality control specifications to ensure that the size distribution, morphology, and tetraspanin expression were consistent with EVs (56). The hTERT-immortalized mesenchymal stem cell-derived EVs (product “ATCC SCRC-4000-EXM”) were obtained in four vials (100 µL each) and were aliquoted at ~616 µg of protein per vial. Each vial was used for one experiment, and we performed four independent experiments with multiple replicates. For Poly I:C (polyinosinic:polycytidylic acid obtained from Thermo/Fisher Scientific) stimulation, we first tested doses of 20 ng, 100 ng, or 1 µg in this study. We stimulated uninfected cortical neurons with Poly I:C (20 ng) for 24 h, followed by hTERT-MSC-EV for another 24 h for combo groups. Similar treatments followed by infection with ZIKV (at a multiplicity of infection [MOI] of 5) were performed for the infected group. Exosome production/release inhibitor (GW4869) was obtained from Santa Cruz Biotechnologies, Inc., and Zika virus (Puerto Rico strain; PRVABC59) used in this study was received from BEI resources. After overnight plating, cortical neurons were first incubated with 1–3 µL of hTERT-MSC-EV equivalent to concentrations of 6.16–18.5 µg protein for 24 h. Neurons were then infected with ZIKV (an MOI of 5). At 96 and 150 h post-ZIKV infection, neurons were assessed for cell viability (by MTT assay) and any cytopathic effects (by imaging neuronal cells for any morphological changes). Furthermore, neurons were pre-treated with 5 µM of the exosome inhibitor GW4869 for 4 h, followed by hTERT-MSC-EV treatment for an additional 24 h, and then infected with ZIKV for 96 or 150 h. Neurons were collected and processed for qRT-PCR or immunoblotting analyses.

### Neuronal cell viability by MTT assay and imaging of cortical neurons

The viability of cortical neurons after treatment with hTERT-MSC-EV and ZIKV infection was determined by an MTT assay as described (16). Briefly, neurons were seeded at a density of 1 × 10^4^ cells/well in a 96-well plate. After overnight plating, neurons were treated with hTERT-MSC-EV (1 µL of hTERT-MSC-EV equivalent to a concentration of 6.16 µg of protein) for 24 h, followed by ZIKV infection for an additional 96 or 150 h. At 96 and 150 h post-infection, the media were removed, and 90 µL of fresh medium was added into each well, followed by 10 µL of MTT solution. The plate was wrapped with aluminum foil and incubated at 37°C for 3 h, and thereafter, 100 µL of MTT solvent and DMSO were added to the wells, respectively. Plates were incubated for another 15 min at 37°C, and absorbance was determined at an OD of 560 and 650 nm. Differences in values determined the cell viability numbers, with the higher absorbances indicating increased viability of neurons. Furthermore, microscopic images of hTERT-MSC-EV-treated/untreated and ZIKV-infected/uninfected neurons at 96 and 150 h were obtained using BioTek’s Cytation 7 imaging multimode reader system (Agilent Technologies). Magnification of 20× was used in all imaging analyses. Scale bar indicates 200 µm. We manually counted the soma/cell bodies of cortical neurons from three independent images obtained as different fields of interest for each group. The average of soma counting from three images was plotted and quantified.

### RNA extractions, cDNA synthesis, and qRT-PCR analysis of cortical neurons

Total RNA was extracted from cortical neurons using Aurum Total RNA Mini Kit, following the protocol from the manufacturer (Bio-Rad, USA). RNA concentrations were measured using BioTek’s Cytation 7 imaging multimode reader system (Agilent Technologies, USA). The cDNA was synthesized using the iScript cDNA Synthesis Kit (Bio-Rad, USA). The qRT-PCR was performed with iQ-SYBR Green master mix and by using the CFX Opus instrument (Bio-Rad, USA), as previously described (16). Primers (for ZIKV, IFN-alpha, IFN-beta, TNF-alpha, actin, and GAPDH) are from our published studies (16, 57–60), and other primers were designed in this current study using the Primer3 online software (https://primer3.ut.ee/). The primer sequences are as follows: BAX (forward, *5′- CTACAGGGTTTCATCCAG-3*′; reverse, *5′-CCAGTTCATCTCCAATTCG-3*′)*,* Caspase 3 (forward, *5′-GAGCAGCTTTGTGTGTGTGA-3*′; reverse, *5′-GGCAGGCCTGAATGATGAAG-*3′), Caspase 9 (forward, *5′- ATGCTCCGTGTCCATTGAGA-3*′; reverse, *5′-AGTCACTGTCCAAGGTCCTG-3*′), and BCL-2 (forward, *5′- TGTGGCCTTCTTTGAGTTCG-3*′; reverse, *5′-TCAGAGACAGCCAGGAGAAA-3*′). The qRT-PCR data were analyzed by the relative standard curve method, in which 10-fold serial dilutions were used to establish standards ranging from 1 to 10^5^ ng. Accordingly, viral loads or gene transcripts were normalized to either mouse beta-actin or total RNA concentrations.

### Immunoblotting analysis

Following hTERT-MSC-EV treatments and ZIKV infection for 96 or 150 h, total lysates from primary cultures of cortical neurons were collected in RIPA lysis buffer containing complete protease and phosphatase inhibitor cocktails. Total protein amounts were measured using the BCA assay kit (Pierce/ThermoScientific Inc., USA), and equal amounts of total protein from whole lysates were used for each group. Total protein (20–25 µg) for each sample was resolved onto 12%–15% SDS-PAGE gels. Gels were blotted onto nitrocellulose membranes and blocked with 5% milk dissolved in 1× TBST buffer (with 0.01% Tween-20). Blots were incubated with anti-ZIKV NS2B primary antibody (1:1,000 dilution; GeneTex, detects a band size of 12 kDa), followed by incubation with goat anti-rabbit HRP-conjugated IgG secondary antibody as 1:5,000 dilution (Boster Bio, Inc., USA). Blots were incubated with detection reagents from the WesternBright ECL kit and imaged using the Chemidoc MP imaging system (Bio-Rad, USA). Immunoblots collected from three independent batches of cortical neuronal and hTERT-MSC-EV experiments were evaluated for densitometric analysis using Bio-Rad imager and software. Band intensities were calculated using ImageJ, and significance was calculated by a statistical method. Total protein profile gel images served as loading controls.

### Statistical analyses

The data sets were analyzed using GraphPad Prism 6 software (San Diego, CA, USA) and Microsoft Excel (Microsoft Corporation). The unpaired, two-tailed *t*-test or one-way ANOVA with Tukey’s *post hoc* multiple comparison tests was used for all analyses. Mean ± SD was used to plot the graphs shown, and statistical significance was considered as *P* < 0.05. All data points represent the biological replicates.

## RESULTS

### Treatment with hTERT-immortalized mesenchymal stem cell-derived extracellular vesicles increases survival of ZIKV-infected cortical neurons

**Fig 1:**
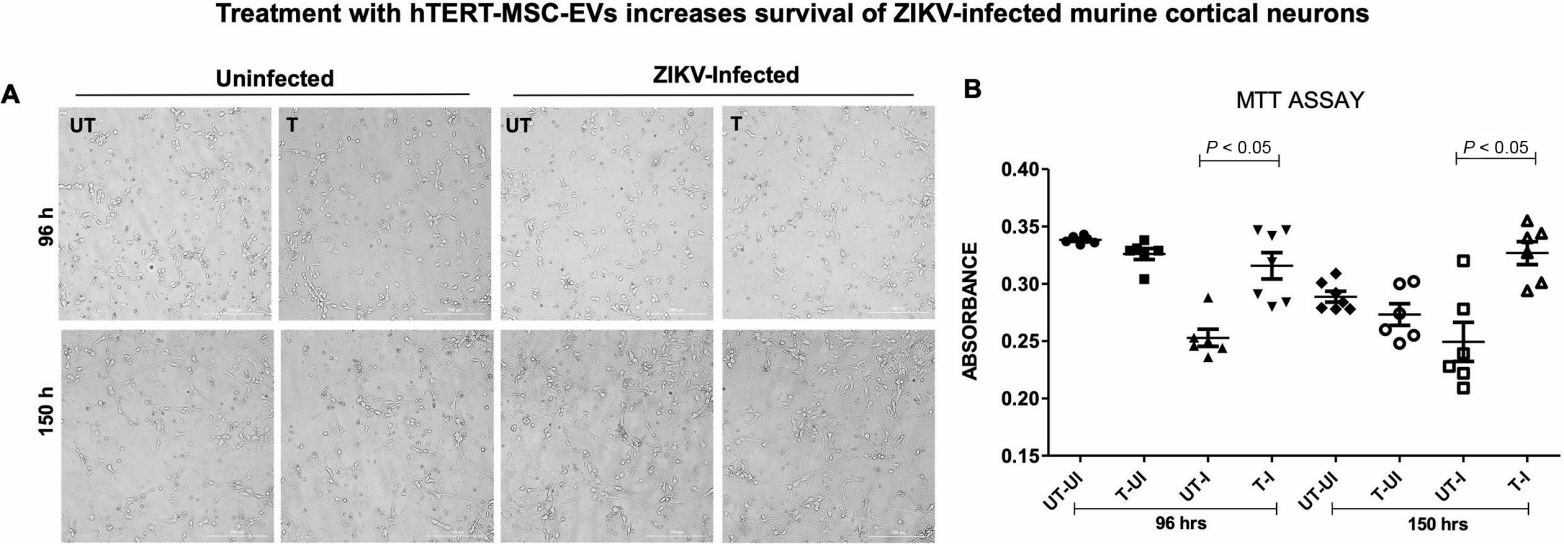
Treatment of hTERT-MSC-EV increases survival of ZIKV-infected cortical neurons. (**A**) Microscopic images showing the morphology of murine cortical neurons treated with non-infectious human mesenchymal stem cell-derived extracellular vesicles for 24 h, followed by ZIKV infection (at an MOI of 5) for 96 or 150 h, respectively. Magnification of 20× was used in all imaging analyses. Scale bar indicates 200 µm. (**B**) MTT assay showing cell viability of cortical neurons incubated with 1 µL of hTERT-MSC-EV for 24 h, followed by ZIKV infection (at an MOI of 5) for 96 and 150 h, respectively. Uninfected neurons and ZIKV-infected neurons, either untreated or treated with hTERT-MSC-EV, are shown as controls. Closed circles/rhombuses denote untreated and uninfected groups (UT-UI) at 96 or 150 h, closed squares/open circles show treated and uninfected groups (T-UI), closed triangles/open squares represent the untreated and ZIKV-infected groups, and closed inverted triangles/open triangles denote treated and ZIKV-infected groups, respectively. All treatments had six to eight independent replicates. *P* value less than 0.05 is considered statistically significant.

To test the effects of hTERT-MSC-EV, we treated primary cultures of murine cortical neurons with hTERT-MSC-EV for 24 h, followed by ZIKV (with an MOI of 5) for 96 or 150 h post-infection (p.i.). An independent ZIKV-infected group was kept as an untreated control. Furthermore, as internal controls, uninfected neurons were either treated with hTERT-MSC-EV or kept as untreated groups. Representative images from each group are shown for comparison and from a field of interest (Fig. 1A). We did not observe any morphological changes or effects in cortical neurons, nor did we find any visual phenotypes upon hTERT-MSC-EV treatment of neurons. Since neurons are non-dividing cells, we do not expect any changes in cell propagation; however, we noted a higher or denser neuronal networking upon ZIKV infection at 150 h p.i. in comparison to 96 h p.i.. To determine whether treatment of hTERT-MSC-EV has any effect on cell viability in cortical neurons upon ZIKV infection, we performed an MTT assay, which showed no significant differences in cell viability between uninfected neurons that were either untreated or treated with hTERT-MSC-EV and collected at 96 or at 150 h post-incubations or treatment (Fig. 1B). However, we noted a significant increase in cell viability of hTERT-MSC-EV-treated neurons infected with ZIKV (T-I) compared to the untreated neurons with ZIKV infection (UT-I) and collected at 96 or 150 h (Fig. 1B). Both treated groups (with ZIKV infection at 96 or 150 h) showed significantly higher cell viability when compared to their respective untreated-ZIKV-infected control groups (Fig. 1B). Since cell viability remained unchanged in uninfected groups of neurons (untreated/treated and at both time points of 96 or 150 h), we also considered showing independent graphs with various scales according to the data values (shown in Fig. 1B) and as two group comparisons (Fig. S1A through D). MTT assay showed a lower trend in cell viability of uninfected and hTERT-MSC-EV-treated group, but it was not significant when compared to the untreated and uninfected group of neurons (Fig. S1A and B). We noted a significant increase in cell viability of ZIKV-infected cortical neurons treated with hTERT-MSC-EV in comparison to the untreated and ZIKV-infected control group (Fig. S1C and D). In addition, we manually counted the soma (cell bodies) of neurons from all groups (only three images were considered for counting from each group) to reveal neuronal counts upon hTERT-MSC-EV treatment and ZIKV infection in comparison to the untreated and ZIKV-infected control group (Fig. S1E). No significant differences were observed for any group that was either untreated or treated with hTERT-MSC-EV or uninfected or infected with ZIKV (Fig. S1E). These data show that treatment with hTERT-MSC-EV increases cell viability in ZIKV-infected cortical neurons.

### Treatment with hTERT-MSC-EV downregulates apoptotic gene expression in ZIKV-infected cortical neurons

**Fig 2:**
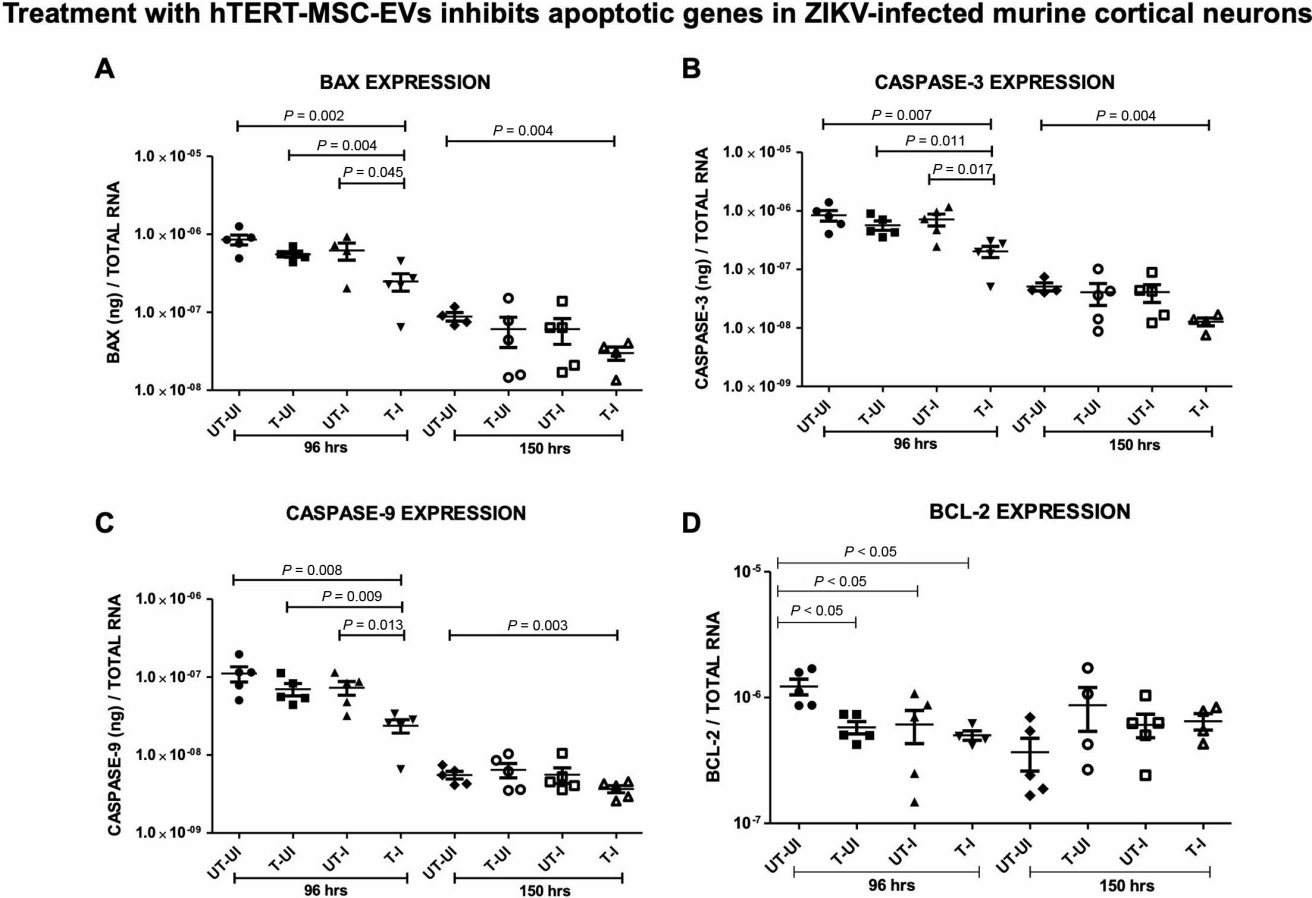
Effects of hTERT-MSC-EV and ZIKV infection on apoptosis marker expression in cortical neurons. The qRT-PCR analysis showing gene expression of apoptotic markers in murine cortical neurons incubated with hTERT-MSC-EV (for 24 h) followed by ZIKV infection (an MOI of 5) for 96 or 150 h. Gene expression of BAX (**A**), Caspase 3 (**B**)**,** Caspase 9 (**C**), and Bcl-2 (**D**) is shown for all groups. Closed circles/rhombuses denote untreated uninfected (UT-UI), whereas closed squares/open circles represent treated uninfected (T-UI) groups, respectively. Closed triangles/open squares indicate untreated infected and closed inverted triangles/open triangles show treated and ZIKV-infected groups, respectively. Transcript levels for each gene were normalized to total RNA amounts, respectively. All treatments had five independent replicates. *P* value less than 0.05 is considered statistically significant.

To understand the increase in cell viability of hTERT-MSC-EV-treated ZIKV-infected cortical neurons, we analyzed the gene expression of apoptotic markers. As expected, we found no significant differences in the gene expression of *bax*, *caspases 3/9,* and *bcl-2* in the uninfected group of cortical neurons at 96 h, whether they were untreated or treated with hTERT-MSC-EV (Fig. S2A through D). An increasing trend was noted in the uninfected group of neurons treated with hTERT-MSC-EV, but no significant differences were noted in comparison to the untreated and uninfected controls (Fig. S2A through D). At 96 h post-ZIKV infection, we noted a significant (*P* > 0.05) reduction in the transcript levels of pro-apoptotic/anti-apoptotic markers such as *bax*, *caspases 3/9,* and *bcl-2* in hTERT-MSC-EV-treated and ZIKV-infected cortical neurons (T-I) when compared to the levels noted in untreated and ZIKV-infected control group (UT-I) of neurons (Fig. S2E through H). All three pro-apoptotic transcript levels (*caspases 3/9* and *bcl-2*) at 96 h were significantly (*P* > 0.05) reduced in hTERT-MSC-EV-treated cortical neurons infected with ZIKV (T-I) compared to the untreated and ZIKV-infected (UT-I) control group of neurons (Fig. 2A through C; Fig. S2E through H). While this effect was pronounced at 96 h tested time point, the modulation was not that effective at 150 h in hTERT-MSC-EV-treated and ZIKV-infected cortical neurons (T-I) (Fig. 2A through D). Although at 150 h, we noted a downregulated trend in the transcript levels of *bax* and *caspases 3/9* in hTERT-MSC-EV-treated and ZIKV-infected group of neurons compared to the untreated and ZIKV-infected group, no significant differences were noted (Fig. 2A through C). In addition, we did not observe any significant (*P* > 0.05) differences in the transcript levels of *bax*, *caspases 3/9,* and *bcl-2* in uninfected neurons upon treatment with hTERT-MSC-EV compared to the levels noted in uninfected and untreated control group at both 96 and 150 h post-treatment (Fig. 2A through D; Fig. S2A through D). At 150 h, and in all tested groups (uninfected or ZIKV-infected and MSC-EVs-treated or untreated), we observed a decreasing trend in the expression of *bax* and *caspases 3/9* compared to the gene expression noted at 96 h. This decreasing trend showed only significant differences between the uninfected and untreated group to the hTERT-MSC-EV-treated and ZIKV-infected group of cortical neurons (Fig. 2A through D). In addition, we found that transcript levels of housekeeping genes such as actin and GAPDH were also altered at both 96 and 150 h tested time points (Fig. S3A and B). The gene expression data for pro-apoptotic/apoptotic markers correlated with increased cell viability observed in hTERT-MSC-EV-treated and ZIKV-infected group. Taken together, these data indicate that treatment with hTERT-MSC-EV perhaps inhibits cell death to increase the cell viability in cortical neurons upon ZIKV infection by downregulating the apoptotic gene expression.

### hTERT-MSC-EV play a key role in modulating ZIKV infection of cortical neurons

**Fig 3:**
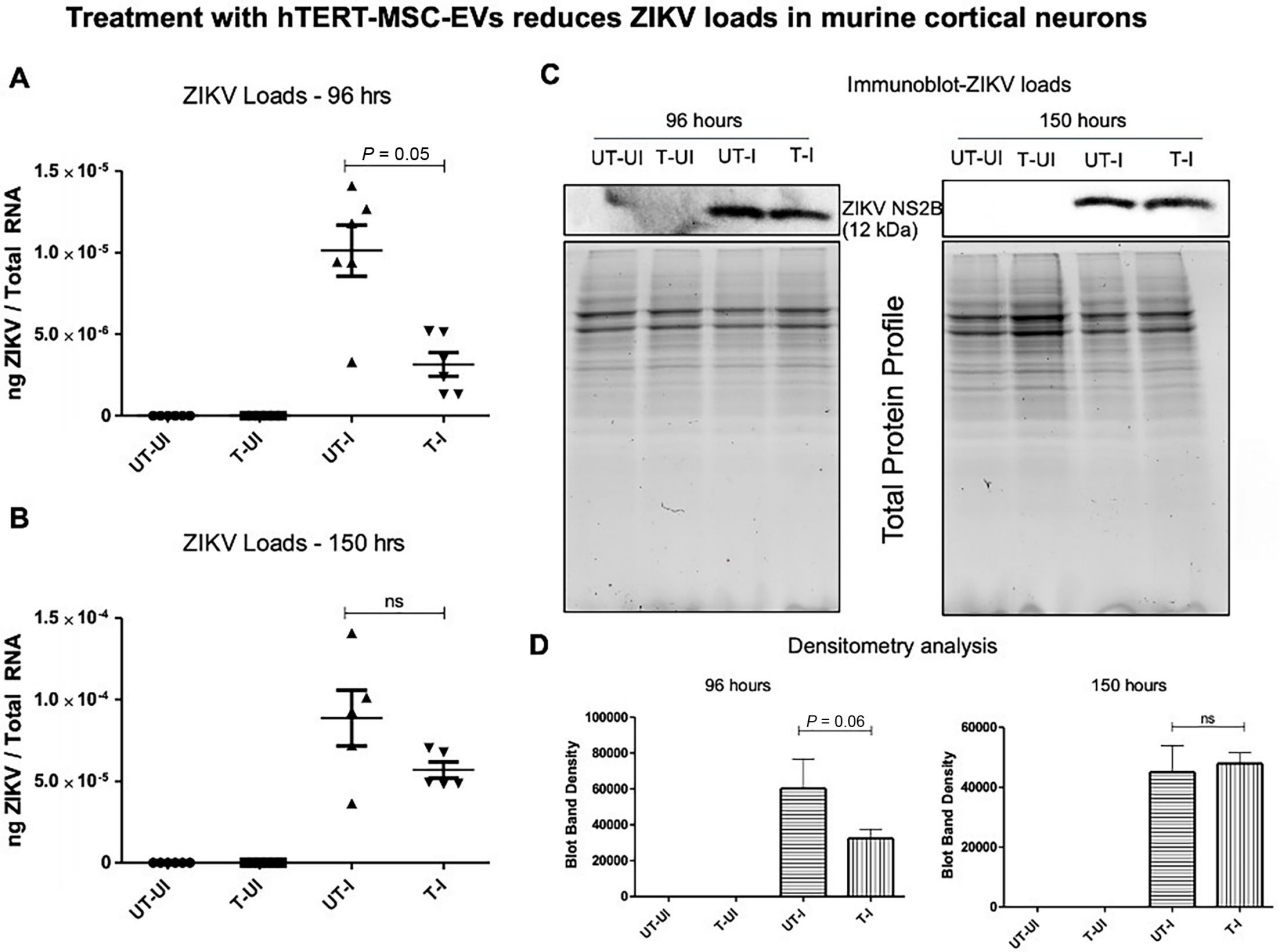
Treatment with hTERT-MSC-EV reduced ZIKV loads in primary cultures of cortical neurons. The qRT-PCR analysis showing ZIKV NS5 transcript levels in cortical neurons incubated with hTERT-MSC-EV for 24 h, followed by ZIKV infection (an MOI of 5) at 96 h (**A**) or 150 h (**B**). ZIKV RNA levels were normalized to total RNA amounts. All treatments had six independent replicates. *P* values less than 0.05 are considered statistically significant. (**C**) Immunoblotting analysis showing ZIKV NS2B protein levels from 96 and 150 h time points. Protein sizes are indicated in kilodaltons (kDa), and the protein profile gel images serve as loading controls. (**D**) Densitometric analysis of immunoblots in panel **C** and Fig. S4 (for ZIKV NS2B protein at 96 and 150 h) is shown.

We then addressed whether the hTERT-MSC-EV treatment of cortical neurons has any effect on ZIKV loads. The qRT-PCR data showed significant (*P* < 0.05) reduction in ZIKV RNA levels (NS5 transcripts) at 96 h post-infection in cortical neurons treated with hTERT-MSC-EV compared to the levels noted in infected and untreated control group of neurons (Fig. 3A). At 150 h, we noted no significant differences for ZIKV loads in cortical neurons treated with hTERT-MSC-EV compared to ZIKV-infected neurons with no treatments (Fig. 3B). As expected, no viral RNA loads were detected in uninfected samples collected at 96 or 150 h post-treatment. In addition, we found no changes in ZIKV load data at 96 or 150 h by determining the copy numbers (Fig. S4A and B). Furthermore, immunoblotting analysis revealed a reduction in ZIKV NS2B protein levels at 96 h post-infection. At the 96 h tested time point, nearly three- to four fold lower levels of ZIKV NS2B protein amounts were detected in hTERT-MSC-EV-treated and ZIKV-infected group (T-I) compared to the levels noted in untreated and ZIKV-infected (UT-I) control group of neurons (Fig. 3C). We observed no differences in ZIKV NS2B protein levels between hTERT-MSC-EV-treated and infected neurons compared to the untreated and ZIKV-infected control group at 150 h (Fig. 3C; Fig. S5). Similar observations were noted in all independent immunoblotting experiments (performed with different batches of neurons and hTERT-MSC-EV treatments) at 96 and 150 h post-ZIKV infection (Fig. S5A through F). Total protein profile gel images (at 96 and 150 h) serve as loading controls (Fig. 3C; Fig. S5A through F). The densitometry data collected from three independent immunoblotting analyses revealed a decrease in viral NS2B protein levels upon hTERT-MSC-EV treatment of cortical neurons at 96 h, but no significant differences were noted at both 96 and 150 h post-ZIKV infection (Fig. 3D). Overall, these data show that hTERT-MSC-EV treatment significantly reduced ZIKV loads in cortical neurons.

### hTERT-MSC-EV treatment increased interferon-beta and decreased TNF-alpha gene expression in ZIKV-infected cortical neurons

**Fig 4:**
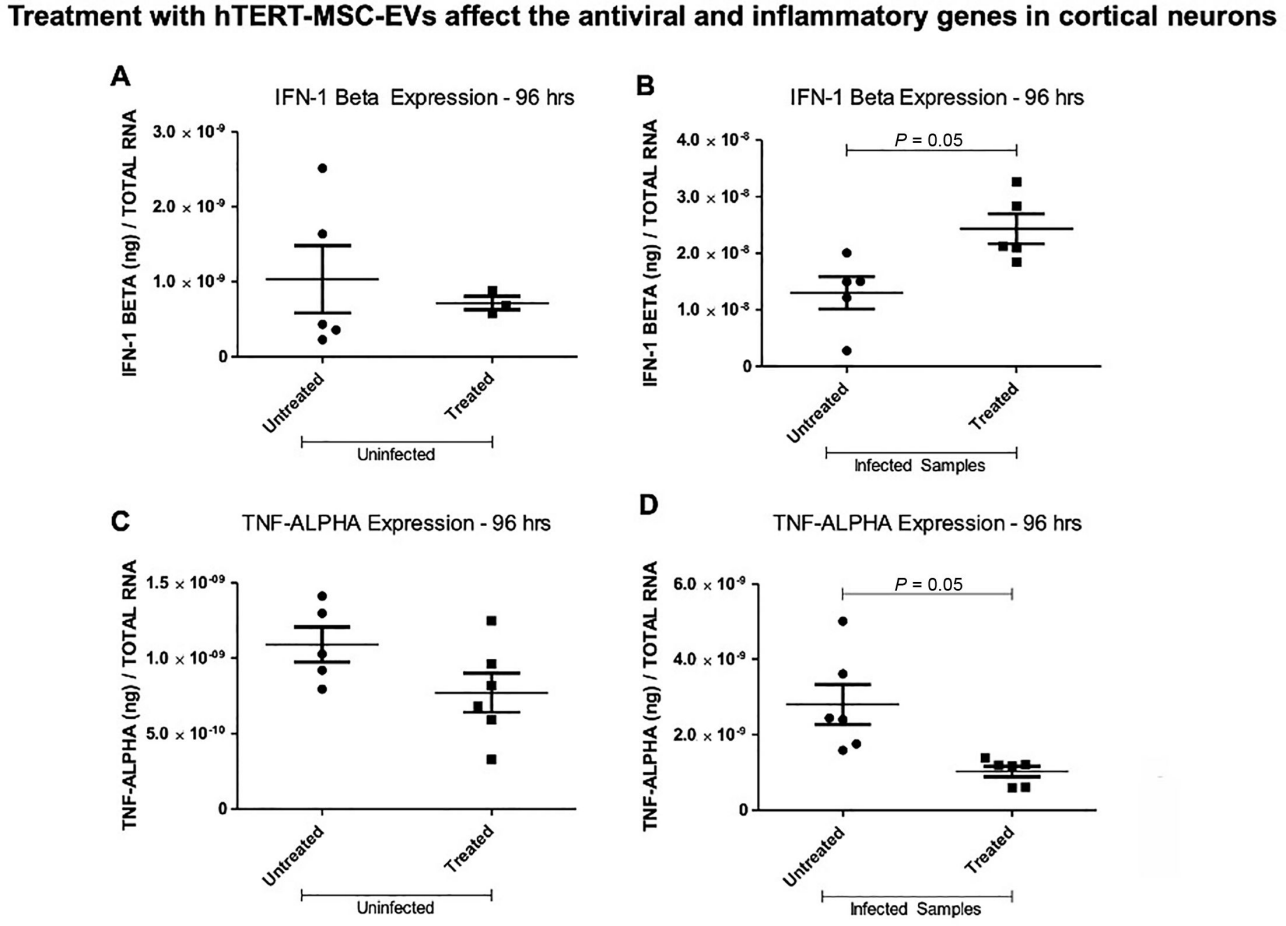
hTERT-MSC-EV-treated and ZIKV-infected cortical neurons modulate immune gene expression. The qRT-PCR analysis showing IFN-1 beta (**A and B**) and TNF-alpha (**C and D**) transcript levels in cortical neurons that are either untreated or treated with hTERT-MSC-EV at 96 h post-ZIKV infection (**B and D**) or uninfected groups (**A and C**). Closed circles denote untreated uninfected or untreated ZIKV-infected groups, and closed squares represent treated uninfected or treated and ZIKV-infected groups, respectively. IFN-1 beta and TNF-alpha transcript levels were normalized to total RNA amounts. All treatments had six independent replicates. *P* values less than 0.05 are considered statistically significant.

To understand the reason for decreased viral protein loads, we determined the expression of immune genes such as IFN-beta and TNF-alpha. IFN-beta plays an important role in the early line of defense against viral infections (61, 62), and TNF-alpha stimulates a downstream signaling cascade related to cell viability (63). The qRT-PCR analysis revealed significantly (*P* < 0.05) increased levels of interferon beta transcripts (Fig. 4A and B) and significantly (*P* < 0.05) reduced levels of tumor-necrosis factor-alpha transcript levels (Fig. 4C and D) in hTERT-MSC-EV-treated ZIKV-infected neurons when compared to the levels noted in untreated ZIKV-infected control group of cortical neurons at 96 h p.i. (Fig. 4B and D). However, no significant differences in either IFN-beta or TNF-alpha transcript levels were noted in the uninfected group of neurons that were either untreated or treated with hTERT-MSC-EV at 96 h (Fig. 4A through D). These data indicate that reduced viral loads in cortical neurons upon treatment with hTERT-MSC-EV could be due to the increased levels of IFN-beta that would regulate the antiviral response.

### Treatment with hTERT-MSC-EV reduced ZIKV loads upon poly I:C stimulation in infected cortical neurons

**Fig 5:**
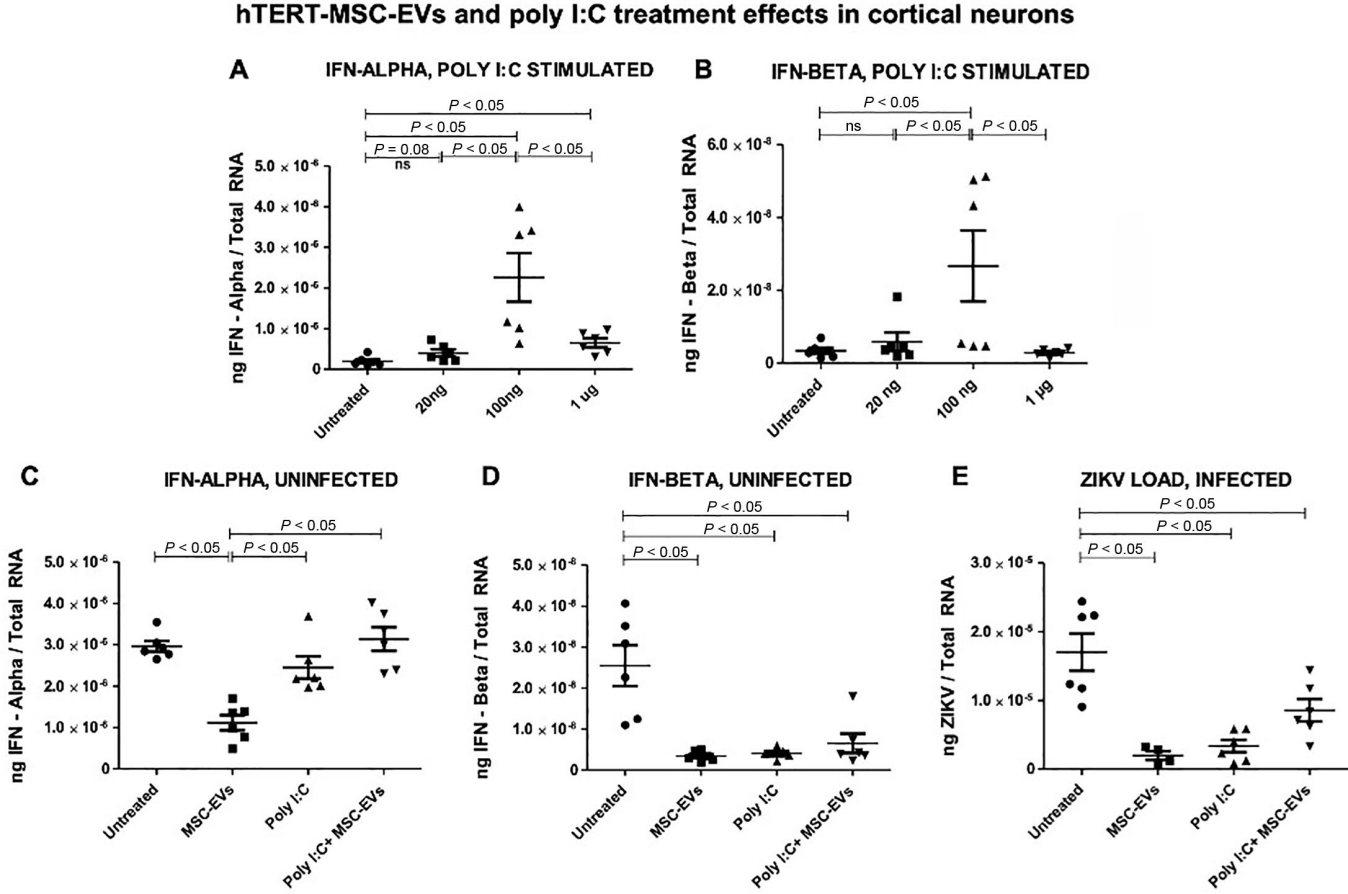
Antiviral response and ZIKV infection upon Poly I:C stimulation and treatment with hTERT-MSC-EV in cortical neurons. The qRT-PCR analysis showing IFN-alpha and IFN-beta (**A and B**) transcript levels in uninfected cortical neurons that are either untreated or treated with Poly I:C (with doses of 20 ng, 100 ng, and 1 µg) for 24 h post-treatment. Gene expression of IFN-alpha (**C**) and IFN-beta (**D**) is shown in uninfected cortical neurons that are either untreated or treated with hTERT-MSC-EV alone or stimulated with Poly I:C alone or in combination (of both hTERT-MSC-EV treatment and Poly I:C stimulation). The qRT-PCR analysis showing ZIKV loads in infected cortical neurons at 96 h post-infection (**E**) that are either untreated or treated with hTERT-MSC-EV alone or stimulated with Poly I:C alone or in combination (of both hTERT-MSC-EV treatment and Poly I:C stimulation). Closed circles denote untreated groups, closed squares represent hTERT-MSC-EV-treated group, closed triangles indicate Poly I:C-stimulated group, and closed inverted triangles show the combo group, respectively (**A–E**). IFN-alpha, IFN-beta, and ZIKV-NS5 transcript levels were normalized to total RNA amounts. All treatments had six independent replicates. *P* values less than 0.05 are considered statistically significant, and ns represents not significant.

Poly I:C is a synthetic double-stranded RNA (dsRNA) analog structurally similar to dsRNA and acts as an endosomal Toll-like receptor 3 interactor/agonist in macrophages, dendritic cells, and B cells (64, 65). It is an immunostimulant used in the form of its sodium salt to simulate viral infections and is a common tool in scientific research on the immune system and antiviral response (64, 66–68). To understand the specificity and effects of hTERT-MSC-EV on interferon expression in uninfected neurons or on inhibition of ZIKV infection in infected neurons, we included stimulation with Poly I:C molecule. First, we tested the doses (with 20 ng, 100 ng, and 1 µg) of Poly I:C and analyzed the expression of interferon-alpha and IFN-beta in uninfected cortical neurons (Fig. 5A and B). We found that in uninfected cortical neurons, both IFN-alpha and IFN-beta had significantly higher expression at 100 ng treatment of Poly I:C, when compared to the untreated or 20 ng or 1 µg treated groups (Fig. 5A and B). Treatment with 1 µg of Poly I:C showed reduced expression of IFN-alpha and IFN-beta in comparison to the dose of 100 ng (Fig. 5A and B). In addition, we show that treatment with hTERT-MSC-EV in uninfected cortical neurons reduced IFN-alpha and repeatedly decreased IFN-beta transcript levels compared to the untreated uninfected group of neurons (Fig. 5C and D). Poly I:C stimulation alone showed no significant differences in IFN-alpha transcripts, but expression levels of IFN-beta levels were significantly reduced compared to the untreated group of uninfected cortical neurons (Fig. 5C and D). Treatments with Poly I:C and hTERT-MSC-EV (in combination) showed a significant increase in IFN-alpha levels compared to the group treated with hTERT-MSC-EV alone, but no significant changes were noted compared to the untreated group of uninfected neurons (Fig. 5C and D). We found a diminished or blunted antiviral response in the Poly I:C stimulation and hTERT-MSC-EV-treated combination group with significantly reduced expression levels of IFN-beta compared to the untreated group of uninfected cortical neurons (Fig. 5C and D). Interestingly, we noted that Poly I:C stimulation and hTERT-MSC-EV treatment as a combination had significantly reduced ZIKV loads compared to the untreated infected cortical neurons (Fig. 5E). These data showed that irrespective of Poly I:C stimulation and ZIKV infection, hTERT-MSC-EV are still efficient in reducing viral loads in infected cortical neurons.

### Treatment with GW4869 inhibitor further reduced hTERT-MSC-EV-mediated ZIKV loads

**Fig 6:**
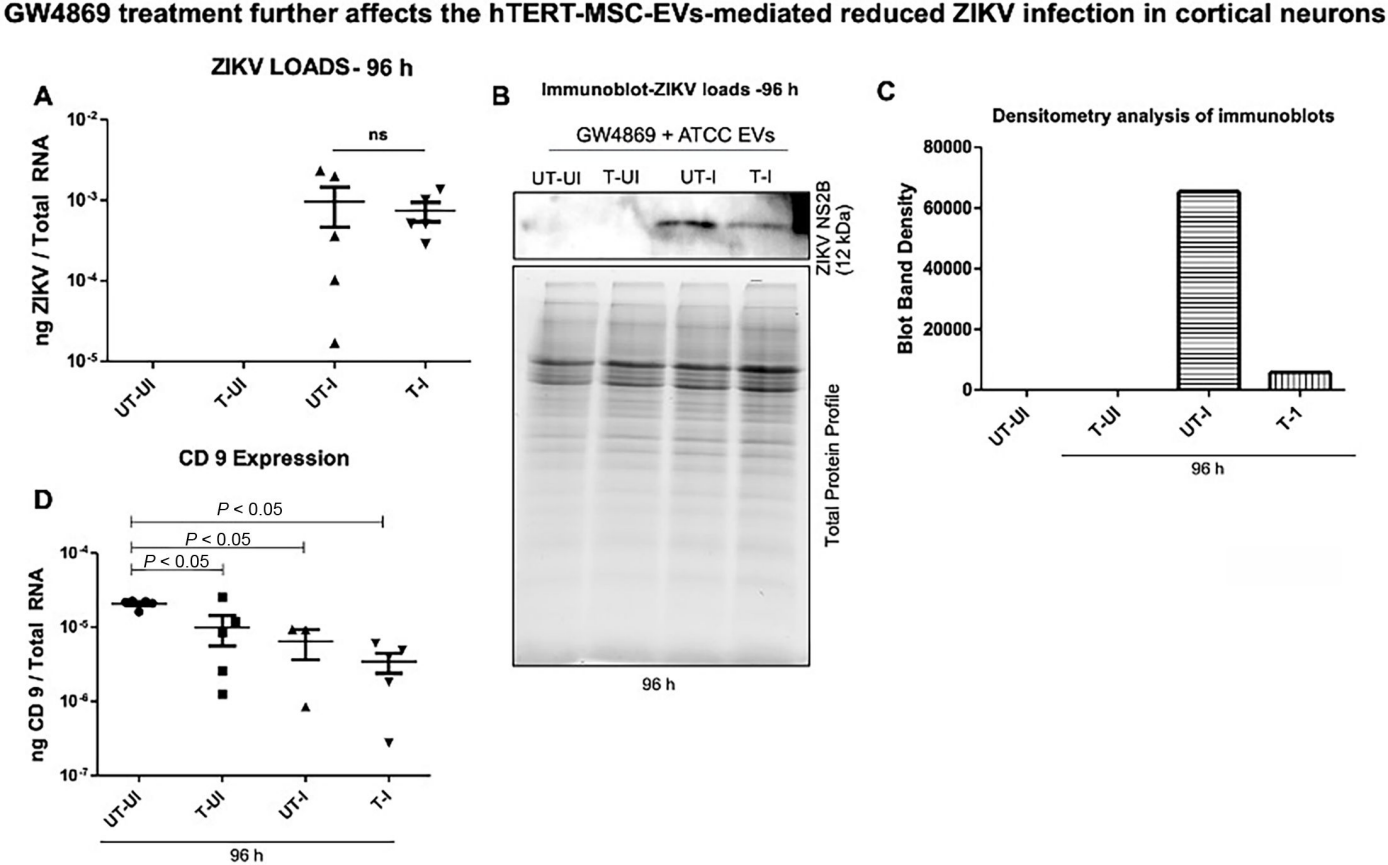
GW4869 and hTERT-MSC-EV treatment reduced ZIKV infection in murine cortical neurons. (**A**) The qRT-PCR analysis showing ZIKV NS5 transcript levels in cortical neurons treated with GW4869 for 4 h, followed by hTERT-MSC-EV treatment for additional 24 h and then infected with ZIKV for another 96 h. (**B**) Immunoblot showing viral NS2B protein in cortical neurons treated with GW4869 (for 4 h), followed by treatment with hTERT-MSC-EV for 24 and 96 h post-ZIKV infection. The size of the viral NS2B protein is indicated in kDa, and the protein profile gel image serves as the loading control. (**C**) Densitometric analysis for immunoblots shown in panel **B** (for ZIKV NS2B protein). (**D**) The qRT-PCR analysis showing expression of CD9 in uninfected and ZIKV-infected groups treated with GW4869 (for 4 h) and treatment with hTERT-MSC-EV (for 24 h), followed by ZIKV infection for 96 h. Transcript levels were normalized to total RNA amounts in both panels (**A and D**), respectively. All treatments had five independent replicates. For statistical significance, *P*-value less than 0.05 was considered, and ns represents not significant.

Our previous studies indicated the importance of exosomes in viral transmission, dissemination, and survival (7, 16, 69). We hypothesized that reduced ZIKV loads could be due to a lower number of cortical neuron-derived EVs presented upon hTERT-MSC-EV treatment. We reasoned that the presence of exogenous MSC-EVs may interfere with the intracellular EV production and release in cortical neurons. We first treated cortical neurons with the GW4869 inhibitor (a pharmacological agent that blocks the production and release of EVs) for 4 h. We then treated these neurons with hTERT-MSC-EV for another 24 h followed by ZIKV infection for 96 h. We observed no significant differences in NS5 transcript levels between the ZIKV-infected groups (untreated/treated) at 96 h p.i. (Fig. 6A). In addition, estimation of copy numbers showed no significant differences between ZIKV-infected and untreated/hTERT-MSC-EV-treated groups (Fig. S6A). However, the ZIKV NS2B protein levels were remarkably lower in hTERT-MSC-EV-treated and ZIKV-infected (T-I) group when compared to the levels observed in untreated- ZIKV-infected (UT-I) group (Fig. 6B). As expected, no NS2B protein was detected in uninfected control groups (either untreated/treated) (Fig. 6B). Total protein profile gel image serves as a loading control (Fig. 6B). Densitometry data correlated with the immunoblotting analysis and revealed a dramatic decrease in viral NS2B protein levels upon GW4869 and hTERT-MSC-EV treatment at 96 h post-ZIKV infection (Fig. 6C).

Furthermore, qRT-PCR analysis also showed that exosomal marker CD9 gene expression was reduced in treated and uninfected (T-UI), untreated-ZIKV-infected, or treated-ZIKV-infected groups compared to the untreated and uninfected (UT-UI) control group (Fig. 6D). No significant reduction was noted between the other groups (Fig. 6D). In addition, we analyzed the expression of exosomal marker CD9 (tetraspanin domain containing transmembrane protein) transcript levels at both 96 and 150 h. In comparison to the 96 h time point, we noted a significant (*P* < 0.05) increase in CD9 transcript levels at 150 h and between the groups of treated and uninfected (96 and 150 h) or untreated and infected (96 and 150 h) or treated and infected (96 and 150 h), respectively (Fig. S6B). Although an increasing trend was observed between the untreated and uninfected and treated and uninfected or untreated and infected and treated and infected groups (at both the 96 and 150 h time points), we found no significant differences in CD9 transcript levels (Fig. S6B). These data show that CD9 expression is upregulated at 150 h compared to 96 h, and both hTERT-MSC-EV treatment and ZIKV infection support this CD9 induction. Our previous studies further indicated that treatment with GW4869 affects the viral loads in both arthropod and mammalian cells (7, 9, 14, 16, 69, 70). We therefore investigated the effect of GW4869 inhibitor on hTERT-MSC-EV treatment and ZIKV infection. Overall, our data show that treatment of cortical neurons with hTERT-MSC-EV increases cell viability, possibly by inhibiting apoptotic gene expression and reducing ZIKV infection, or increasing the antiviral response and affecting EVs-mediated transmission.

## DISCUSSION

The clinical applications of EVs, emphasizing them as biomarkers in diagnostics and therapeutics, are rapidly advancing in the recent years (5, 6, 15, 18, 71–80). Exosomes have been reported to play key roles in mediating viral infections of the central nervous system (CNS), which could be the cause of neurological diseases (81, 82). The reparative and regenerative properties of stem cell-derived EVs in the context of various CNS pathologies have been emerging very fast (21–39). Several studies have reported the therapeutic effects of mesenchymal stem cell-derived EVs (21–40). These studies analyzed the proteomics and miRNA profiles of exosomes derived from human embryonic stem cells and human-induced pluripotent stem cells (21–40). In the current study, we observed that treatment of murine cortical neurons with “ATCC SCRC-4000-EXM,” hTERT-immortalized MSC-EVs, showed no cytopathic effects or morphological changes at both tested time points of 96 and 150 h post-ZIKV infection. The manual counting of neuronal cell bodies or soma from three different images for each group did not show any differences. However, the MTT assay determining the neuronal cell viability showed that treatment with hTERT-MSC-EV significantly increased the cell viability of cortical neurons infected with ZIKV at 96 and 150 h post-infection. These data suggest that hTERT-MSC-EV treatment renders neuroprotection and enhances the survival of ZIKV-infected cortical neurons compared to the untreated neurons with ZIKV infection. Furthermore, compared to hTERT-MSC-EV-treated infected neurons, the untreated/treated uninfected neurons showed no significant changes, thus suggesting a secure use of hTERT-MSC-EV in medical applications. Furthermore, we were able to correlate the increased cell viability of ZIKV-infected cortical neurons with a decrease in cell apoptotic markers such as BAX, caspase 3, caspase 9, and Bcl-2. At 96 h post-ZIKV infection, these markers were significantly reduced in hTERT-MSC-EV-treated neurons in comparison to the untreated and ZIKV-infected group of neurons. In addition, ZIKV-infected cortical neurons treated with hTERT-MSC-EV showed significantly reduced apoptosis marker levels compared to the untreated/treated uninfected groups, thereby suggesting a potential effect of hTERT-MSC-EV in specifically inhibiting apoptosis upon ZIKV infection. At 150 h post-ZIKV infection, the BAX and caspase 3/9 levels were lower in hTERT-MSC-EV-treated groups compared to other groups. Both increased cell viability and reduced gene expression of cell death markers in hTERT-MSC-EV-treated and ZIKV-infected group indicate the supporting scenario of neuronal protection. Our data align with the recently published study that revealed the hTERT-MSC-EV reduce apoptosis and regulate cell cycle machinery in damaged retinal epithelial cells (56). These data reveal that treatment of cortical neurons with hTERT-MSC-EV inhibits apoptosis, thus correlating with increased cell viability upon ZIKV infection. Taken together, these analyses revealed that treatment of murine cortical neurons with hTERT-MSC-EV increases cell viability upon ZIKV infection, and this increase correlates with reduced expression of apoptosis markers.

We noted significantly decreased NS5 transcripts and reduced viral NS2B protein loads in ZIKV-infected cortical neurons treated with hTERT-MSC-EV compared to the untreated and ZIKV-infected group of neurons. Three independent immunoblots performed from different batches of neurons and treatments consistently showed a reduction in NS2B viral protein levels at 96 h in the group of ZIKV-infected neurons treated with hTERT-MSC-EV when compared to the untreated and ZIKV-infected group. These data showed consistency in ZIKV reduction at 96 h and not at 150 h of infection. Densitometry analysis pooled from three independent immunoblot analyses showed a three- to four fold decrease in NS2B protein loads at 96 h across all three independent immunoblots. This suggests neuroprotection due to reduced viral infection in neurons treated with hTERT-MSC-EV. It is noteworthy that at 150 h, viral NS2B loads were not reduced in hTERT-MSC-EV-treated-ZIKV-infected neurons compared to the hTERT-MSC-EV-treated-ZIKV-infected neurons at 96 h. This may be due to the decreasing effects of hTERT-MSC-EV over the longer time point of 150 h and the increasing levels of CD9 that might support the neuronal EV-mediated viral transmission from neuron to neuron. In addition, the amount of hTERT-MSC-EV treatment is only 6.16–18.5 µg per well (which is a very small amount). Perhaps the use of higher concentrations might provide longer neuroprotection against ZIKV infection of cortical neurons. We believe that the treatment with hTERT-MSC-EV is fading at 150 h as it is a longer incubation time. Moreover, we are not aware of the mechanism for hTERT-MSC-EV uptake and viability. Since EVs fuse with recipient cells, we believe that hTERT-MSC-EV also fuse with neurons to deliver the content. However, we are not aware if the content delivered inside the host recipient cells remains active and functional after 150 h. Our future studies will delineate all these points to enhance this line of investigation. Between the hTERT-MSC-EV-treated and untreated groups, there is no significant difference at 150 h, which could be due to the lower number of neurons (due to increased death with ZIKV infection) in the untreated group. We assume that increased cell viability might not be the cause for reduced viral loads detected in hTERT-MSC-EV-treated group of ZIKV-infected neurons at 96 h, as no differences were noted in the total protein profiles of cortical neuronal cells. However, we did not observe any increase in cell viability of uninfected neurons with hTERT-MSC-EV treatment or in the untreated group, which suggests an interdependent effect of cell viability and ZIKV infection upon hTERT-MSC-EV treatment. Our data show that treatment with hTERT-MSC-EV reduces the viral loads. We believe that it is hTERT-MSC-EV-mediated neuronal protection that increases the cell viability to control viral loads.

We believe that the increase in IFN-beta expression upon ZIKV infection could be a host-mediated effect, which is perhaps modulated by hTERT-MSC-EV treatment upon recognition of infection. We did not find higher IFN-beta expression in uninfected neurons that were either untreated or treated with hTERT-MSC-EV, thus suggesting that it is important for hTERT-MSC-EV to recognize viral infections to support the enhancement of antiviral response. This blunted or diminished expression of IFN-beta in uninfected cortical neurons indicated that the antiviral response is mediated by independent host signaling. It can also be assumed that in these uninfected neurons, treatment with hTERT-MSC-EV may not support the increase in IFN-beta response due to the absence of ZIKV infection. These data also indicate that hTERT-MSC-EV recognize the ZIKV infection and support the enhanced IFN-beta expression as an antiviral response in infected neurons. We repeated these IFN-beta expression analyses along with IFN-alpha expression upon treatment with hTERT-MSC-EV and found a reproducible expression pattern with significantly reduced levels of IFN-beta as well as IFN-alpha in uninfected cortical neurons. In addition, the group with Poly I:C stimulation (at 20 ng) alone showed significantly reduced IFN-beta expression compared to the untreated group, but no significant differences were noted with IFN-alpha expression when compared to the untreated group of uninfected cortical neurons. These data suggest that Poly I:C alone at this dose of stimulation is not efficient in enhancing the antiviral response. The combination treatment of Poly I:C and hTERT-MSC-EV also showed significantly reduced IFN-beta expression compared to the untreated group. However, IFN-alpha showed a significant increase in the combination group compared to the hTERT-MSC-EV-treated group but not to the untreated group. These data suggest that this blunted or diminished expression of IFN-alpha and IFN-beta in hTERT-MSC-EV-treated or Poly I:C alone stimulated groups is due to either no infection or lower amounts of Poly I:C stimulation. We avoided an exaggerated amount (100 ng) of Poly I:C to recognize the effects and efficiency of hTERT-MSC-EV. The increased IFN-alpha expression in the combination (poly I:C and hTERT-MSC-EV treatment) group compared to the hTERT-MSC-EV alone treated group suggests that hTERT-MSC-EV recognize the presence of Poly I:C and enhance the antiviral response in uninfected cortical neurons. Our finding that the proinflammatory response by reduced TNF-alpha expression is perhaps directly regulated by hTERT-MSC-EV treatment. Furthermore, significantly reduced ZIKV infection in hTERT-MSC-EV treatment alone or in combination group with Poly I:C stimulation further supports that hTERT-MSC-EV inhibit ZIKV replication and viral infectivity and may similarly protect neurons against other viral infections. These data clearly suggest that interferon-beta expression is induced upon viral infection and is perhaps not an effect of hTERT-MSC-EV treatment.

To not exclude the possibility that endogenously produced EVs by cortical neurons may bounce back the viral infection and transmission, we analyzed and found increased CD9 (exosomal enriched marker) transcript levels at 150 h compared to 96 h time point. To further test the effects of the role of hTERT-MSC-EV, we treated cortical neurons with GW4869, an exosome release inhibitor. Although no differences were noted in NS5 gene transcripts (at 96 h), dramatically reduced NS2B protein loads in hTERT-MSC-EV-treated ZIKV-infected neurons were evident compared to the loads noted in untreated and ZIKV-infected control group. The densitometry analysis revealed a six- to seven fold reduction in NS2B levels upon GW4869 treatment. These data suggest that GW4869 is not only effective on neuronal exosomes but also in controlling ZIKV-mediated viral transmission and performs better in combination with hTERT-MSC-EV treatment. We have previously shown that GW4869 treatment dramatically reduces ZIKV infection and viral transmission in murine cortical neurons and that this effect was effective from 24 to 96 h post-infection (16). The reduction in CD9 transcripts further corroborated the effects of GW4869 and hTERT-MSC-EV combination treatments. Reduced CD9 transcript levels upon GW4869 and hTERT-MSC-EV treatments did correlate with the reduced viral protein loads in hTERT-MSC-EV-treated ZIKV-infected neurons at 96 h. We propose a model that elucidates the role of hTERT-MSC-EV in promoting neuronal cell viability, inhibiting apoptosis, and reducing ZIKV infection and exosome-mediated transmission (Fig. S6C). In addition, hTERT-MSC-EV could affect exosomal markers that promote exosome biogenesis. Reduced ZIKV protein loads could be a result of downregulated exosome biogenesis, and viral transmission/dissemination via neuronal EVs may be an indirect effect of hTERT-MSC-EV treatment. Overall, these data suggest a potential therapeutic effect of hTERT-MSC-EV in enhancing cell viability of ZIKV-infected murine cortical neurons, inhibiting apoptosis, and reducing the viral infection and exosome-mediated transmission. Our data indicate that hTERT-MSC-EV treatment decreases the exosomal marker CD9, affecting exosome biogenesis and resulting in decreased viral infection that could be further inhibited by GW4869 treatment.

## Data Availability

All data presented in the article will be available from the corresponding author and upon request.

## SUPPLEMENTAL MATERIAL

Supplemental material spectrum.02524-25-s0001.pdf Fig. S1 to S6.

ASM does not own the copyrights to Supplemental Material that may be linked to, or accessed through, an article. The authors have granted ASM a non-exclusive, world-wide license to publish the Supplemental Material files. Please contact the corresponding author directly for reuse.
