# Supplementary material for: hTERT-immortalized mesenchymal stem cell-derived EV treatment reduces ZIKV-induced cortical neuronal death, infection, and exosome-mediated transmission
Source: Microbiol Spectr. 2026 Apr 13;14(5):e02524-25. doi: 10.1128/spectrum.02524-25 (PMC13141902; doi:10.1128/spectrum.02524-25)
Supplement: Supplemental material — Fig. S1 to S6. [file spectrum.02524-25-s0001.pdf]

SUPPLEMENTARY DATA

hTERT immortalized mesenchymal stem cell-derived EVs treatment reduces ZIKV-induced cortical neuronal death, infection, and exosome-mediated transmission

Kehinde Damilare Fasae [1], Ana Melentijevic Eckert [2], Girish Neelakanta [1], Hameeda Sultana [1, *]

[1] Department of Biomedical and Diagnostic Sciences, College of Veterinary Medicine, University of Tennessee, Knoxville, TN, USA. [2] American Type Culture Collection (ATCC), Gaithersburg, MD, USA

Running title: hTERT-MSC-EVs modulate ZIKV-infected cortical neurons

Key Words: hTERT-MSC, Extracellular Vesicles, cortical neurons, mice, ZIKA virus, neuronal viability, apoptosis, GW4869, CD9

*** Corresponding Author**: Department of Biomedical and Diagnostic Sciences, College of Veterinary Medicine, University of Tennessee, Knoxville, TN 37996, USA, Email: hsultana@utk.edu, Phone: (865) 974-8217.

Supplementary Information File Includes Supplemental- Figures and Figure Legends.

I. **Supplemental Figures and**
II. **Supplemental Figures Legends**

hTERT-MSC-EVs treatment increased cell viability of cortical neurons infected with ZIKV

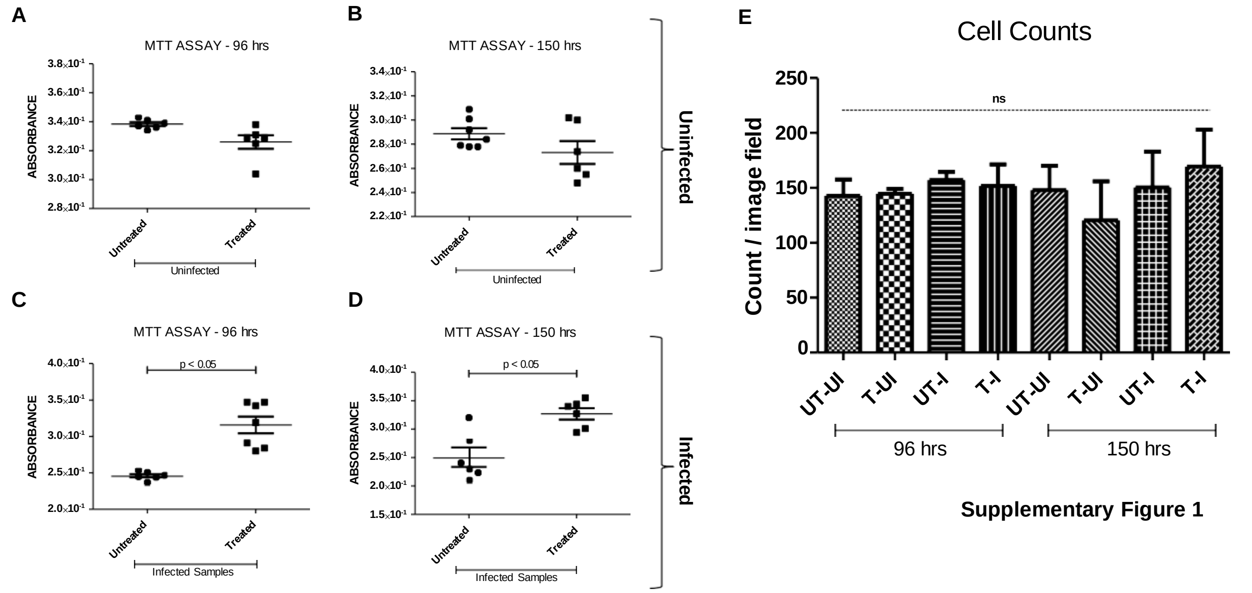

Supplementary Figure 1. Treatment of hTERT-MSC-EVs and ZIKV infection on cortical neurons. (A-D) MTT assay showing cell viability of cortical neurons incubated with 1 µl of hTERT-MSC-EVs for 24 h followed by ZIKV infection (at MOI of 5) for 96 h and 150 h, respectively. Uninfected neurons **(A, B)** and ZIKV-infected neurons **(C, D)** either untreated or hTERT-MSC-EVs-treated groups are shown. Closed circles denote untreated uninfected, or untreated ZIKV-infected groups (**A-D**). The closed squares represent treated uninfected, or treated and ZIKV-infected groups, respectively. (**E**) Manual counts of neuronal soma/cell bodies collected from three independent images taken from random fields of interest for each group is shown. All treatments had 6-8 independent replicates. P value less than 0.05 is considered statistically significant.

Treatment with hTERT-MSC-EVs inhibits apoptotic gene expression in ZIKV-infected murine cortical neurons

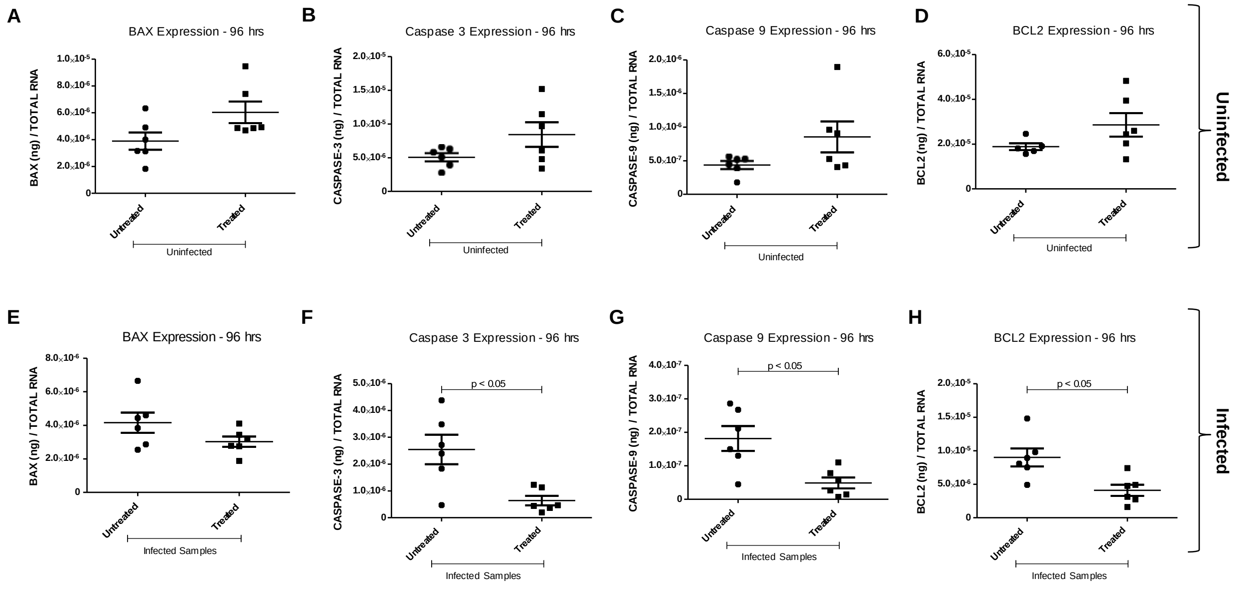

Supplementary Figure 2

Supplementary Figure 2. Treatment of hTERT-MSC-EVs affects apoptotic gene expression in cortical neurons. QRT-PCR analysis showing the gene expression of apoptotic markers (**A-H**) in murine cortical neurons incubated with hTERT-MSC-EVs (for 24 h) followed by ZIKV infection (5 MOI) for 96 h. Gene expression of BAX **(A, E)**, Caspase 3 **(B, F),** Caspase 9 **(C, G)** and Bcl-2 **(D, H)** is shown from uninfected-untreated/treated (**A-D**) or hTERT-MSC-EVs treated/untreated and ZIKV-infected groups (**E-H**). Closed circles denote untreated- uninfected or ZIKV-infected groups, whereas closed squares represent treated- uninfected or ZIKV-infected groups, respectively. Transcript levels for each gene were normalized to total RNA amounts, respectively. All treatments had 6 independent replicates. P value less than 0.05 is considered statistically significant.

Actin and GAPDH gene expression is affected upon hTERT-MSC-EVs treatment

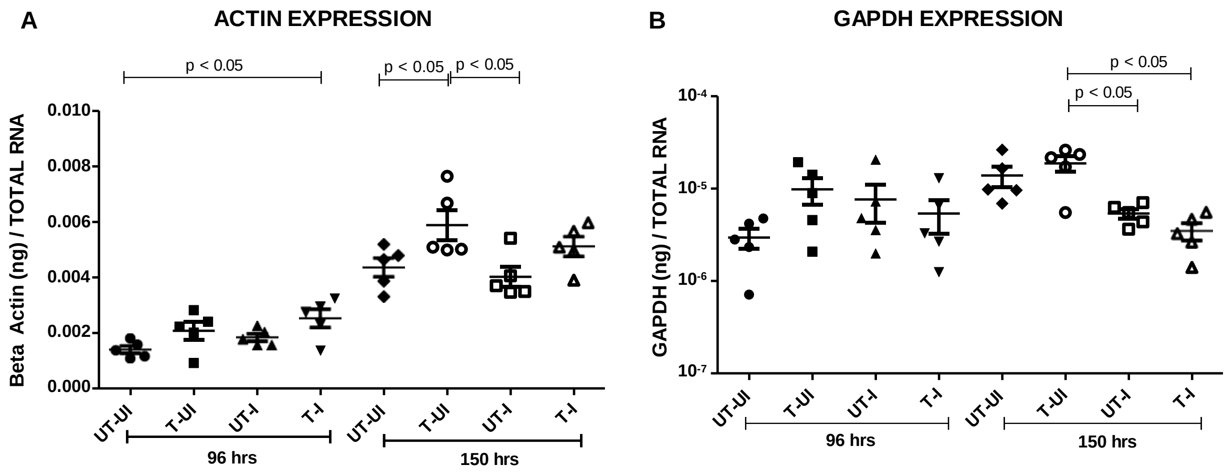

Supplementary Figure 3

Supplementary Figure 3. Actin and GAPDH gene expression is affected upon hTERT-MSC-EVs. QRT-PCR analysis showing transcript levels of actin (**A**) and GAPDH (**B**) genes in cortical neurons treated with hTERT-MSC-EVs (for 24 h) followed by ZIKV infection (5 MOI) for 96 h or 150 h. Transcript levels were normalized to total RNA amounts, respectively. All treatments had 5 independent replicates. P value less than 0.05 is considered statistically significant.

Treatment with hTERT-MSC-EVs reduces ZIKV infection in cortical neurons - at 96 h (copy number data)

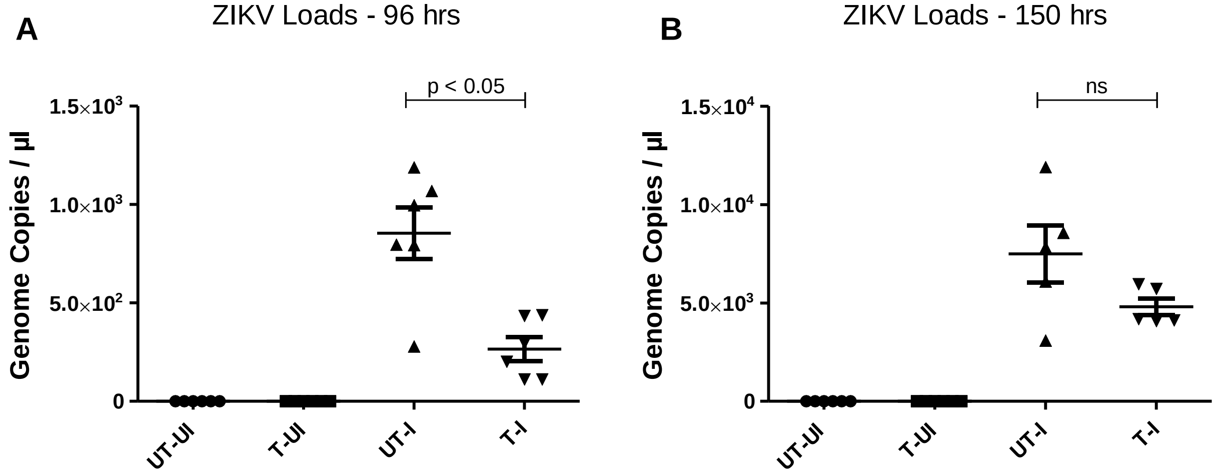

Supplementary Figure 4

Supplementary Figure 4. Treatment with hTERT-MSC-EVs reduced ZIKV loads in primary cultures of cortical neurons (copy number data). QRT-PCR analysis to determine the copy numbers showing ZIKV NS5 transcript levels in cortical neurons incubated with hTERT-MSC- EVs for 24 hours followed by ZIKV infection (MOI of 5) at 96 h (A) or at 150 h (B). All treatments had 6 independent replicates. P values less than 0.05 is considered statistically significant.

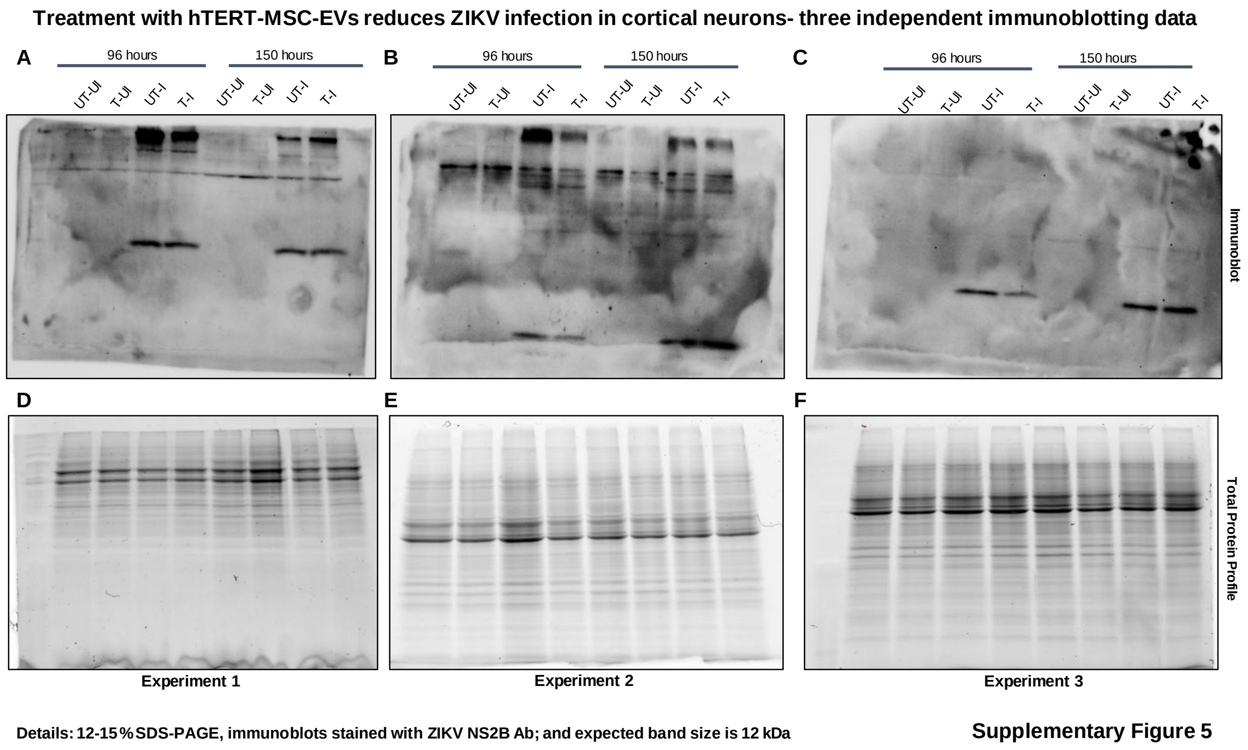

Supplementary Figure 5. Immunoblotting analysis from three independent batches of cortical neurons showed reduced ZIKV loads in hTERT-MSC-EVs. Immunoblots performed on total lysates of cortical neurons that are either uninfected and treated/untreated or hTERT-MSC-EVs treated/untreated and ZIKV infected (at 96 h or 150 post infection) (**A-F**). Immunoblots are shown from three independent batches of neurons (**A-C**) isolated from embryonic brains collected from different mice with same gestational period of 16 days. Complete immunoblots are shown to reveal the NS2B viral protein (12 kDa) indicated with arrow heads. Total protein profile-stained gel images (**D-F**) are shown for loading controls.

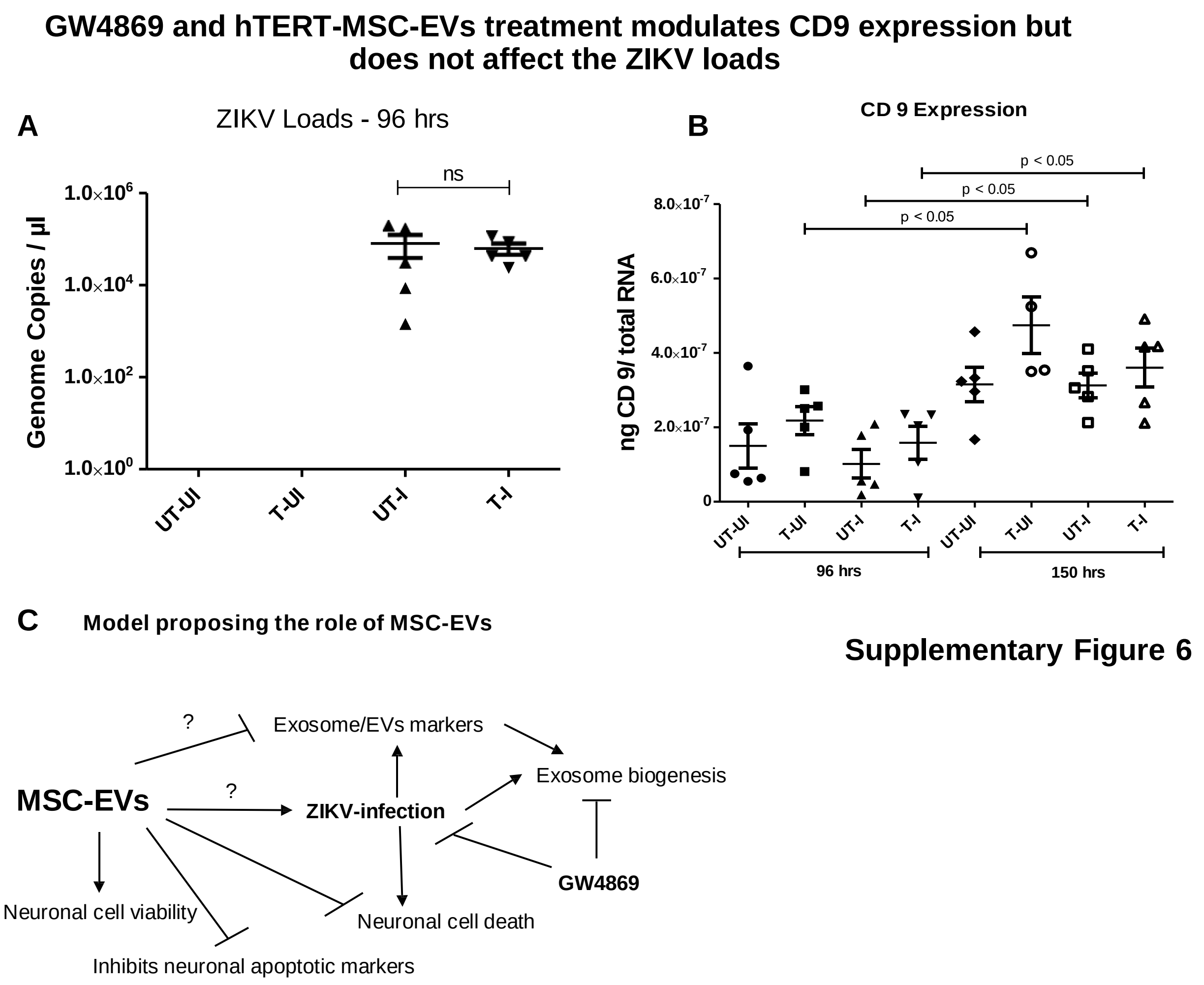

Supplementary Figure 6. Treatment of GW4869 and hTERT-MSC-EVs does not affect ZIKV loads but modulates CD9 expression in ZIKV-infected cortical neurons. QRT-PCR analysis to determine the copy numbers showing ZIKV NS5 transcript levels in cortical neurons incubated with GW4869 and hTERT-MSC-EVs for 24 h followed by ZIKV infection (MOI of 5) at 96 h **(A)**. QRT-PCR analysis showing CD9 expression **(B)** in murine cortical neurons treated with hTERT-MSC-EVs (for 24 h) followed by ZIKV infection (5 MOI) for 96 h or 150 h. Transcript levels were normalized to total RNA amounts, respectively. All treatments had 5 independent replicates. P value less than 0.05 is considered statistically significant. **(C)** Model proposing the role of hTERT-MSC-EVs in cortical neurons is shown. The model elucidates the

role of hTERT-MSC-EVs in promoting neuronal cell viability, inhibiting apoptosis and reducing ZIKV infection and exosome-mediated transmission.
